# 

**Published:** 1852-07

**Authors:** 


### PHILADELPHIA COLLEGE OF DENTAL SURGERY.

We have just received the first annual announcement of this school. We are glad to see they have organized under such favorable auspices, and hope to see the school well sustained. We should suppose that the City of Philadelphia, turns out yearly, not less than ten to twenty Dental Practitioners, and should of itself give ten or twelve students yearly to the College.

Drs. White, Townsend and Arthur, are already well known to the profession, and we should think would fill their respective Chairs with ability. The other gentlemen although not as well known to us, we have no doubt, are well qualified for their posts.

The College has a charter from the Pennsylvania Legislature. The lectures commence on first Monday of November, and close first of March.

| | |
|---|---|
| Fees—each Professor's ticket, - - - - - - | $15 00 |
| Demonstrator's ticket, - - - - - - - - - | 10 00 |
| Matriculation paid once, - - - - - - - - - | 5 00 |
| Diploma fee, - - - - - - - - - - - - | 30 00 |

The following is the organization of the Faculty:

J. D. White, M. D., D. D. S., Professor of Anatomy and Physiology.

Ely Parry, M, D., D. D. S., Professor of Chemistry, Materia Medica, and Special Therapeutics.

Robert Arthur, D. D. S., Professor of Principles of Dental Surgery.

Elisha Townsend, M. D., D. D. S., Professor of Operative Dental Surgery.

T. L. Buckingham, M. D., Professor of Mechanical Dentistry.

D. P. Whipple, M. D., Demonstrator of Surgical and Mechanical Dentistry.

### NEW YORK COLLEGE OF DENTAL SURGERY AT SYRACUSE.

This Institution was in operation last winter; we have not been informed with what success. Professor A. Westcott, M. D., D. D. S,, formerly Professor of Operative and Mechanical Dentistry in the Baltimore College of Dental Surgery, fills the Chair of Theory and Practice of Dental Surgery and Dental Technology.

The other Chairs are Institutes of Dentistry, Dental Hygiene, and Comparative Anatomy. This Chair has as yet, also been filled we believe by Professor Westcott.

The Chair of Anatomy and Physiology and General Principles of Surgery, is filled by A. B. Shipman, M. D., late Professor of Surgery in the Indiana Medical College.

The Chair of Special Pathology and Therapeutics, is filled by Thomas Spencer, M. D., formerly Professor of Theory and Practice of Medicine in the Geneva Medical College, and late Professor of the same in the Chicago Medical College.

The Chair of Chemistry, is filled by R. E. Stevens, M. D. The Demonstrator of Mechanical and Surgical Dentistry, is Daniel Van Denburgh, D. D. S.

The Faculty appears to have been well selected, and Professor Westcott, is well and favorably known to the Dental Profession.

### OHIO COLLEGE OF DENTAL SURGERY.

We will give a very brief synopsis of the condition and prospects of this Institution. It has now been in operation over seven years, during which time about forty have obtained by a regular course of study, and evidence of qualification, the honors of the Institution. Of this number all with very few exceptions, are now in lucrative practice, and generally in very desirable situations. The College Buildings have been purchased by an Association of Stockholders, and over one-third, some $1,400 the first payment on the property, has been paid in. The Lecture Room, the Anatomical Rooms, the Laboratory, &c., excepting the Infirmary Room and Museum, were constructed when put up with special reference to the wants of the school, and need no alteration. The Infirmary and Museum Rooms, were originally constructed for school purposes, and these the Association design rebuilding so as to give a beautiful front to the College edifice; this will be not less than three story, and will be arranged for male and female Infirmary, Museum, and private Dissecting Rooms.

At the last meeting of the Stockholders, a committee was appointed to get plans and bids for building, and report at next meeting, which will be at commencement.

A fund has been appropriated for the purpose of meeting this outlay. This is obtained by the interest on the stock which the Faculty pay. This improvement which we hope will he worthy of the profession, will be made in one or two years at most. If the Alumni of the College unite with the Stockholders, as they have manifested a desire to do, the Dental College will be an honor to the State and Queen City. The mode of instruction is such as we think the wants of the profession most directly point out; the object being to qualify the student both theoretically and practically for the duties of the profession.

The Faculty last year appropriated a yearly fund for the purchase of books, and such instruments and apparatus as may be needed for the Institution. This fund, will in a few years, secure all the dental publications in the English language, and such works on Anatomy, Pathology, Physiology and Chemistry, as may be needed. We think we are justified in saying, that the Faculty are more desirous of establishing the Institution on a permanent and prosperous footing than for any special self-aggrandizement.

We are much pleased to notice, in the announcement of the Medical College of Ohio, that the Chairs vacated at the close of their last session, have been filled by men of the first class of talent, learning, and distinction. The Chair of Theory and Practice, was resigned by Professor John Bell, and that of Surgery by Professor R. D. Mussey; to the first of which the distinguished Professor, Daniel Drake, has accepted the appointment; and Professor Baxley, the able lecturer on Anatomy in the Institution for the past two years, has been transferred to the position left by the venerable Professor Mussey; and the Chair of Anatomy has been offered to, and accepted by, Professor Cobb, of Louisville, whom the West has long been proud to acknowledge as one of her profoundest Anatomists.

We think we risk nothing in saying, that with such a combination of talents as is found in the present Faculty, (Drake, Cobb, Baxley, Locke, Lawson, and others,) together with the splendid new edifice just completed, the College will, in the future, enjoy large and annually increasing classes of students, and that the "Alumni" of the School will give in the future a higher tone and a more dignified position to the Medical Profession in the West.

We have from our exchanges, several interesting Medical Journals replete with useful and instructive matter.

The first we will notice is the Western Lancet, edited by Professor L. M. Lawson and George Mendenhall, M. D. This is a journal of about seventy pages, issued monthly, and is a fair exponent of the principles of Medicine and Surgery in Cincinnati. Its original matter is abundant and well chosen. Its selected articles from foreign and other journals, are judiciously culled, with a view to meet the wants of the Western Medical Profession; and its Editorials and criticisms, are spicy, impartial, and independent, in vindication of the true principles of science. There is no Western Medical Journal that we can more confidentially recommend to the support of the profession.

*The present No. of the Register.*—This is the last No. of Vol. 5, and we would take this occasion to thank our many friends in the profession who have encouraged us in our labors. Our other duties have generally been so pressing, that we could not devote as much time to the preparation of the Register as we should like.

We are more and more convinced of the need of such a work in the West, and we are more satisfied that a work of the kind can be sustained. But we believe more easily with the money, than with good and able contributors. But few of our profession in the West, are in the habit of writing for any Periodical. A few write occasionally; but we are sorry to say, none regularly and systematically. This we regard as almost necessary to make a work of the kind always interesting.

We are satisfied there are those well able to do this, and we have hopes for the future. The profession and individual advancement require it, and it should be accomplished. If those of age and experience will not write, the younger ones should, they should form this habit, and the earlier the better.

*Our Subscribers.*—We have always considered those who have been in receipt of the Register for more than two or three Nos., as regular subscribers, and we shall send all such bills for the amount due. We much prefer if the Register is not wanted, to have it sent back, and so marked that we may know where and from whom it is returned. This can be done through the Postmaster without any expense to us. There are some in arrears for two, three, and even four volumes. We must attribute this to carelessness and not to any intention to get the paper without paying for it. We hope on the receipt of this No., and the bills therewith, we shall get a remittance from all who are in arrears, and thus be enabled to present to the Society at our next meeting, clean books.

*The Mississippi Valley Association of Dental Surgeons,* holds its Eighth Annual Meeting in the Ohio College of Dental Surgery, Cincinnati, on second Wednesday of September next, at 10 A. M. We hope the members will be all in attendance.

*The American Society of Dental Surgery,* hold their next Annual Meeting at Newport, R. I., on the first Tuesday of August, 1852.

Drs. M. Rogers, and Jesse W. Cook, have both resumed the practice of Dental Surgery in this city.

### RECEIPTS FOR DENTAL REGISTER, VOL. 5.

Dr. B. A. Satterthwaite, Lima, O.
" H. C. Howell, Hamilton, O.
" M. N. Manlove, Logansport, Ia.
" Merchant Kelly, Bentonville, Ia.
" T. R. Willard, Dayton, O.
" John McClure, Carrolton, Miss.
" E. Bray, Evansville, Ia.
" John Hugh, Bastrop, Texas.
" L. M. Cochran, Matagorda, Texas.
" D. W. Harris, West Liberty, O.
" James B. Streeter, Allegan, Mich.
" I. Forbes, St. Louis, Mo.
" Wm. S. Jones, Russelville, Ala.
" D. E. Ellis, Hanover, N. Y.
" R. M. Kenedy, Logansport, Ia.
" T. L. Bancroft, Point Pleasant, Mo.
" Nichols & Johnson, Indianapolis, Ia.

### INDEX TO DENTAL REGISTER OF THE WEST.

### VOL. 5.

ORIGINAL COMMUNICATIONS. PAGE.

Atmospheric Pressure Plates, by Dr. J. S. Ullery, 84
Association of Ohio College, D. S., 164
A Case of Third Dentition, &c., 243
Congenital Absence of Lateral Incisors, 169
Dr. Griffith's Address, 1
" James Taylor's Introductory Address, 65
Dental Irritation, by J. Taft, D. D. S., 87
Dr. Wright's Address to Graduating Class, O. C. D. S., 159
" James Taylor's Communication to A. J. D. S., 148
" Baxter's Condensing Forceps, 170
Letter of Dr. A. Berry, 92
Letter on the insertion and preparation of Natural Teeth, 94
Letter from J. O. Martin, 245
Osseous Union of Teeth, by H. Scott, 91
Proceedings of Mis. V. A. D. S., 6
Practical thoughts on tooth drawing, by C. T. Cushman, D. D. S., 26
Report of the Mis. V. A. D. S,, reviewed by Dr. J. Taylor, 142

SELECTIONS.

A new mode of setting Artificial Teeth, 95
Artistic Dentistry, 97
A New Anæsthetic, 117
Dr Allen's Letter, 181
Dr. Rosting, 183
Dwinelle on Deep Seated Dental Caries, 223
Proceedings Twelfth Annual Meeting Am. S. D. S., 111
" " " " continued, 172
" " " " " 193
Physician's Visiting List, 240
Treatment of Dental Caries, &c., by R. Arthur, D. D. S., 34
World's Fair, 51

### EDITORIAL.

A Trip on the Railroad, 87
American S. D. S., 60
A New Compound for Casts, 187
American Whig Review, 25
College Commencement, 125
Charleston Medical Journal and Review, 25
Dental Times and Advertiser, 59
Dental Colleges, 60
Dental Chair, 124
Dental Recorder, 124
Dental Depot at Louisville, 126
Dr. Allen's Improvement, 184
" Hunter's, " 186
Dr. Hunter's Claims Vindicated by himself, 247
Enlargement of the Register, 60
Extraction of Teeth, by Dr. Cushman, 62
Fine Gold Fillings, 187
Gardette's Proposition to establish a Lectureship, &c., 119
J. D. Chevalier, 752
New York World's Fair, 59
New York College Dental Surgery, 249
New Dental Periodical, 752
Ohio College Dental Surgery, 125 249
Our Exchanges. 126
Ohio Medical College, 250
Ohio Medical and Surgical Journal, 251
Proceedings of Mis. V. A. D. S., 59 125
Persons claiming to be Graduates of O. C. D. S., 126
Philadelphia College Dental Surgery, 248
Present No. of the Register, 253
Receipts for Register, 189 254
Review of Remarks on Professional Dental Education, &c., 250
Society Dental Surgeons State of N. Y., 60
Specimens for Museum, O. C. D. S., 62
Stockholders O. C. D. S., 125
To Our Subscribers, 60 259
Treatment of Dental Caries, 60
The College Library and Museum, 126
To Subscribers of Dental Register, 126
The Meetings of Am. Society D. S., and the Mis. V. A. D. S., 253

